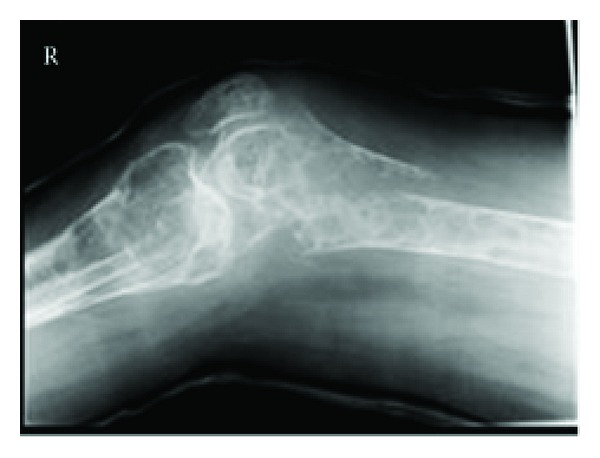# Nasu-Hakola Syndrome: An Unusual Cause of Pathological Fractures

**DOI:** 10.1155/2012/817189

**Published:** 2012-01-19

**Authors:** Jaykar R. Panchmatia, Natasha Jiwa, Neil Soneji, John Paul Murphy

**Affiliations:** ^1^Trauma and Orthopaedic Surgery, Hillingdon and Mount Vernon Hospitals, Rickmansworth Road, Northwood, London HA6 2RN, UK; ^2^Northwick Park Hospital, London HA1 3UJ, UK

## Abstract

Nasu-Hakola syndrome is a hereditary cause of pathological fractures. Uniquely, patients also develop neuropsychiatric symptoms and signs. The disease is ultimately fatal. We propose a management strategy for pathological fractures in sufferers based on the stage of the disease.

## 1. Introduction

Nasu-Hakola Syndrome (NHS) is a condition characterised by lytic lesions of the bone and an early onset fronto-temporal dementia. The disease typically results in death by the fifth decade of life [1]. To date, around 200 cases of this autosomal recessive disease have been reported, and to the authors' knowledge this is the first documented case in an individual of South Asian origin [2]. The syndrome is of interest to orthopaedic surgeons as the overwhelming majority of patients initially present in the second and third decades of life with symptoms such as wrist and ankle pain and pathological fractures. As such, a timely diagnosis by the treating orthopaedic surgeon can result in the more effective management of this disease [3].

## 2. Case Report

**Figure 1:**
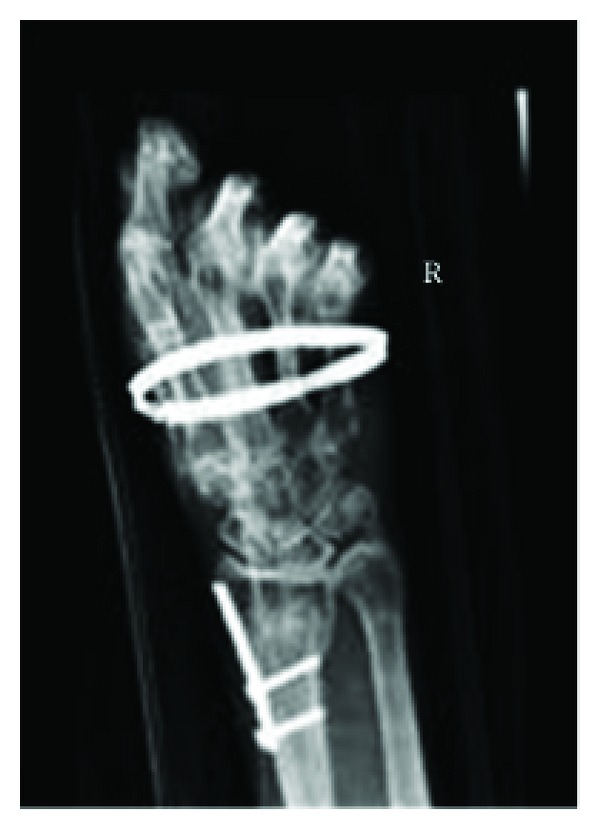


This former civil engineer presented to his local Accident and Emergency department 15 years ago, at the age of 37, with a fragility fracture of the distal radius. The injury was managed with an open reduction and internal fixation (Figure 1). Prior to this admission, the patient was entirely asymptomatic. The next year, he was readmitted to his local hospital having sustained a fragility fracture of the tibial diaphysis following a mechanical fall at home. This was treated conservatively. In the subsequent two years, the patient and his partner noted a subtle decline in his cognition with brief periods of short-term memory loss. Consequently, a cranial MRI scan was performed. The MRI scan revealed sclerosing leukodystrophy and the diagnosis of Nasu-Hakola syndrome established. 

In the ten years that have followed the initial diagnosis of NHS, the patient has experienced a progressive neurological decline. Although initially suffering with isolated antegrade amnesia, he now suffers with tonic-clonic seizures, is unable to communicate, and has no active movement. He requires constant care and has sustained further pathological fractures when, by way of example, being turned in bed. One year ago he sustained a femoral diaphyseal fracture which united following conservative management, and three months ago, he sustained a further femoral diaphyseal fracture on the opposite side which again is being managed conservatively.

## 3. Discussion

NHS, also referred to as polycystic lipomembranous osteodysplasia with sclerosing leukoencephalopathy, was first described by Terayama in 1961 [2]. It was, however, subsequent research conducted by Nasu in Japan and Hakola in Finland that resulted in the disease's frequently used eponym [2]. NHS is an autosomal recessive condition typically affecting the gene DAP 12 located on chromosome 19q13.1. DAP 12 is believed to be expressed in osteoclasts and microglial cells, both of which are derived from a common haematopoietic stem cell population. It is for this reason that patients suffer with a combination of musculoskeletal and neurological symptoms and signs.

The disease can be regarded as having four distinct phases: (1) latent, (2) osseous, (3) early neuropsychiatric, and (4) late neuropsychiatric [4]. Patients will typically present to an orthopaedic surgeon at the second stage of the disease. At this early stage, prior to the onset of neuropsychiatric symptoms, the differential diagnosis includes conditions such as multiple intraosseous lipomata, multifocal cystic angiomatosis, and histiocytosis X. However, with careful radiographic and histological evaluation, each of these alternative diagnoses can largely be excluded. NHS is characterised by the presence of bilateral expansive cyst like lesions separated by septa (Figure 2). Histological specimens sent at the time of surgery will show the cysts to be lipid filled. The presence of associated neuropsychiatric symptoms and signs can be regarded a pathognomonic of NHS. 

The early diagnosis of NHS is key to providing effective treatment as it allows the surgeon to establish a life expectancy for the patient and to tailor the goals of surgery accordingly. Given the markedly reduced life expectancy of sufferers, surgery performed in Stages 2 and 3 often needs to be radical in order to allow early rehabilitation and return to normal daily activities. This may, by way of example, be in the form of a joint arthroplasty to treat intra-articular fractures in young individuals [5]. In stage 4 of the disease, however, individuals are often unable to walk, suffer with protracted seizures and recurrent infections of the urinary and respiratory tract. Consequently, we would argue that on balance the risks of surgery are high and the benefits are minimal in the later stages of the disease. As such pathological fractures sustained at this late stage ought to be managed conservatively. 

## 4. Conclusion

NHS is a debilitating disease typically affecting young adults. A timely diagnosis can be achieved by conducting a detailed analysis of radiology and histology in young patients presenting with pathological fractures. One cannot rely on the ethnicity of a patient, as contrary to popular belief NHS can occur in patients that are not of Northern European or Japanese descent. In the early stages of the disease, pathological fractures ought to be treated aggressively whilst, in the later stages of the disease, we would advocate the conservative management of pathological fractures.

## Figures and Tables

**Figure 2:**